# Selection of Cryoprotectant in Lyophilization of Progesterone-Loaded Stearic Acid Solid Lipid Nanoparticles

**DOI:** 10.3390/pharmaceutics12090892

**Published:** 2020-09-19

**Authors:** Timothy M. Amis, Jwala Renukuntla, Pradeep Kumar Bolla, Bradley A. Clark

**Affiliations:** Department of Basic Pharmaceutical Sciences, Fred Wilson School of Pharmacy, High Point University, High Point, NC 27268, USA; tamis@highpoint.edu (T.M.A.); jrenukun@highpoint.edu (J.R.); pbolla@miners.utep.edu (P.K.B.)

**Keywords:** solid lipid nanoparticles, cryoprotectant, lyophilization, trehalose, surfactants, freezing rate

## Abstract

Cryoprotectants are often required in lyophilization to reduce or eliminate agglomeration of solute or suspended materials. The aim of this study was to select a cryoprotecting agent and optimize its concentration in a solid lipid nanoparticle formulation. Progesterone-loaded stearic acid solid lipid nanoparticles (SA-P SLNs) were prepared by hot homogenization with high speed mixing and sonication. The stearic acid content was 4.6% *w/w* and progesterone was 0.46% *w/w* of the initial formulation. Multiple surfactants were evaluated, and a lecithin and sodium taurocholate system was chosen. Three concentrations of surfactant were then evaluated, and a concentration of 2% *w/w* was chosen based on particle size, polydispersity, and zeta potential. Agglomeration of SA-P SLNs after lyophilization was observed as measured by increased particle size. Dextran, glycine, mannitol, polyvinylpyrrolidone (PVP), sorbitol, and trehalose were evaluated as cryoprotectants by both an initial freeze–thaw analysis and after lyophilization. Once selected as the cryoprotectant, trehalose was evaluated at 5%, 10%, 15%, and 20% for optimal concentration, with 20% trehalose being finally selected as the level of choice. Evaluation by DSC confirmed intimate interaction between stearic acid and progesterone in the SA-P SLNs, and polarized light microscopy shows successful lyophilization of the trehalose/SA-P SLN. A short term 28-day stability study suggests the need for refrigeration of the final lyophilized SA-P SLNs in moisture vapor impermeable packaging.

## 1. Introduction

Solid lipid nanoparticles (SLNs) are nano-sized colloidal carriers (10 nm to 1000 nm) composed of solid lipids, drugs, and surfactants in specific ratios. SLNs provide drug-carrying capability and have several advantages over traditional drug delivery systems [1,2,3]. Of particular benefit is the general biocompatibility of the lipid particle and drug loading capacity, as well as the stability of the nanoparticle and the drug, and the potential for sustained payload release. Additionally, SLNs have the potential to increase bioavailability when dosed orally [4]. Particle size of SLNs has an impact on their cellular uptake and therapeutic index [5], and therefore maintenance of particle size during processing becomes an important control parameter.

SLNs can be prepared using a variety of methods, including high shear homogenization, hot homogenization, cold homogenization, ultrasonic or high-speed homogenization, solvent emulsification/evaporation, microemulsion-based, supercritical fluid, spray drying, and double emulsion [3,6]. The method of preparation is in large part driven by the characteristics of the lipid and the drug to be encapsulated.

Solid lipids that may be chosen for formulating SLNs include fatty acids, mono-, di-, and triglycerides, cationic lipids, and waxes [7]. Melting temperature of lipids, solubility of drug in lipid, and polymorphic behavior of crystalline lipid have all been considered in choice of lipid or lipid system [6]. Surfactants are used to modify surface tension and solubility of the drug in the lipid, as well as to modify the surface characteristics of the solidified SLNs. Surfactants that have been used in the preparation of SLNs include phosphatidyl choline, soy and egg lecithin, poloxamer, poloxamine, polysorbate 20, polysorbate 80, sorbitan monolaurate, sorbitan monooleate, sodium dodecyl sulphate, tyloxopol, sodium oleate, sodium taurocholate, sodium glycocholate, and butanol [6,8]. The surfactant system may be selected based on the particle size and polydispersity index (PI) of the resulting nanoparticles, as well as drug entrapment efficiency [9]. The hydrophile–lipophile balance (HLB) technique has been used to ensure the proper ratio of surfactants are used with the lipid selected [10,11].

In the processes that include dispersion of lipids in water, once rendered solid SLNs are isolated by centrifugation and/or lyophilization. In the latter technique, nanoparticles can agglomerate due to the stresses associated with freezing, resulting in a measurable increase in particle size [12]. This can be modulated by the addition of a cryoprotectant which reduces the intimate contact of lipid particles during the freezing process and protects the particles from significant agglomeration [12,13].

Franzé et al. have shown that the formation of a glassy matrix with extremely high viscosity and low mobility upon freezing (i.e., vitrification) can limit fusion of liposomal vesicles [14]. Similarly, this glassy matrix may immobilize macromolecules, preventing them from interacting [15]. This concept has been extended to nanocapsules as well [16], and in this work it is anticipated that the glassy matrix may prevent the fusion of nanoparticles during freezing. Allison et al. assert that separation of individual particles within the unfrozen phase during freezing prevents aggregation [17]. A cryoprotectant may increase the viscosity and the volume of the unfrozen phase, reducing the likelihood of particle interaction during freezing [17], although in Allison’s description of particle isolation a glassy matrix is not required to be formed during freezing. Therefore, selection of the cryoprotectant and its appropriate concentration becomes an integral part of successful formulation development of SLNs.

The objective of this study was to develop progesterone-loaded stearic acid SLNs and select an appropriate cryoprotectant and its concentration for lyophilization of the final SLN solid.

## 2. Materials

Progesterone, stearic acid, polysorbate 80 (Tween^®^ 80), and trehalose dihydrate were purchased from Acros Organics (Fair Lawn, NJ, USA). Sodium taurocholate was obtained from Biosynth Chemistry and Biology (Itasca, IL, USA). Lecithin, poloxamer 188, sorbitan monolaurate (Span^®^ 20) and d-sorbitol were procured from Alfa Aesar (Tewksbury, MA, USA). Polysorbate 20 (Tween^®^ 20) was purchased from Sigma Aldrich (St. Louis, MO, USA). Kolliphor RH 40 was obtained from BASF (Florham Park, NJ, USA). Mannitol was purchased from J.T.Baker (Phillipsburg, NJ, USA). Polyvinyl pyrrolidone 40 was procured from MP Biomedicals (Solon, OH, USA). Dextran 70 was obtained from Tokyo Chemical Industry (Portland, OR, USA). Glycine as USP powder was purchased from Fischer Scientific (Pittsburgh, PA, USA). HPLC-grade methanol and tetrahydrofuran (THF) were purchased from Fisher Chemical (Fair Lawn, NJ, USA). Ultrapure water (filtered and deionized, Pureflow, Graham, NC, USA) was used for all formulations and analyses.

## 3. Methods

### 3.1. Progesterone Solubility in SA

The solubility of progesterone in stearic acid was determined at 80 °C. Five grams of stearic acid was heated to 80 °C in a controlled temperature water bath under magnetic stirring, followed by the addition of progesterone in approximately 2% *w/w* aliquots. The mixture was observed visually to confirm that the progesterone had dissolved.

### 3.2. Determination of Particle Size, Polydispersity Index and Zeta-Potential

Particle size, PI, and zeta potential were measured using a Beckman Coulter Delsa Nano C Particle Size and Zeta Potential Analyzer (Beckman Coulter, Brea, CA, USA). Samples for particle size analysis were prepared by adding 200 µL of SLNs to 8 mL of deionized water. Samples for zeta potential analysis were prepared by adding 200 µL of SLNs to 12 mL of deionized water. All measurements were performed in triplicate (*N* = 3).

### 3.3. Screening of Surfactants

Lecithin/sodium taurocholate, Poloxamer 188, Koliphor RH 40, and polysorbate 80 were all evaluated as surfactants by measuring particle size, PI, and zeta potential of SLNs formulated with them, respectively. Once a final surfactant/system was chosen, additional evaluation was performed on optimal concentration, again using particle size, PI, and zeta potential.

### 3.4. Formulation of Progesterone Loaded Stearic Acid Solid Lipid Nanoparticles

SLNs were prepared using hot homogenization with high speed mixing and sonication. Five grams of stearic acid were melted at 80 °C in a controlled temperature water bath. Simultaneously, 1 g of lecithin and 1.144 g of sodium taurocholate were heated in 100 mL of deionized water to 80 °C. After the stearic acid was melted, 500 mg of progesterone was added to the stearic acid and allowed to mix under magnetic stirring for 5 min allowing for formation of a clear solution. The surfactant solution was added to the stearic acid/progesterone and homogenized for 10 min at 7000 rpm (Fisher homogenizer model 850, Thermo Fisher Scientific, Waltham, MA, USA). Then the mixture was sonicated with a Fisher brand model FB 705 probe sonicator and model number CL-334 probe at 30 amps for 5 min. Finally, the suspension was suddenly cooled in an ice-water bath until the suspension reached 25 °C (approximately 6 min).

#### 3.4.1. Ultracentrifugation and Optimization

Ultracentrifugation was performed at 15,000 rpm (Relative Centrifugal Force (RCF) = 40,000) using a Hitachi UC CP100NX Ultracentrifuge (Eppendorf Himac Technologies LTD, Hamburg, Germany). To investigate the impact of centrifuging time, four samples of raw suspension were centrifuged for 0, 30, 60, 120, and 180 min. The transmittance at 850 nm (Jenway 6405 UV/Vis spectrophotometer, Cole-Parmer, Staffordshire, UK) was determined for each sample. Transmittance of 850 nm visible light was used to evaluate the concentration of suspended particles after ultracentrifugation, with a higher transmittance indicating a more complete settling of particles.

#### 3.4.2. Particle Washing

The raw nanoparticle suspension was centrifuged for two hours at 15,000 rpm to isolate the wet pellet. The supernatant was then decanted and collected to calculate the unentrapped drug by HPLC analysis. Deionized water was added to each sample of wet pellet. A glass stirring rod was used to dislodge the pellet from the bottom of each sample. The nanoparticles were then re-suspended by vortexing for 1 min and centrifuged again. Finally, these samples were decanted to complete the washing.

### 3.5. Selection of Cryoprotectant

The final cryoprotectant agent was selected from a freeze–thaw study and subsequent lyophilization. Nanoparticles were washed and 7 mL of 10% cryoprotectant solutions of dextran, glycine, mannitol, PVP 40, sorbitol, and trehalose were added to two sample sets of wet pellets. One set of samples was placed on an insulated block (to reduce conductive heat transfer) at −20 °C for 24 h (slow freeze) while the other set was placed directly on metal freezer shelves at −70 °C for 24 h (rapid freeze). Both sample sets were allowed to thaw at room temperature for 24 h after freezing. Particle size analysis was performed after thawing. Following lyophilization, redispersibility was investigated by replacing the amount of water lost during lyophilization, vortexing the suspension for 30 s, and visually observing for ease of dispersion and for gross undispersed particles; this was graded using a 3-level qualitative scale (level 1: fully redispersed within 30 s of vortexing; level 2: a few remaining undispersed visible agglomerates still present after 30 s of vortexing; level 3: very little or no redispersion of solid after 30 s of vortexing). Particle size of the resuspended nanoparticles was measured.

### 3.6. Optimization of Cryoprotectant Concentration

Washed nanoparticles were resuspended with 7 mL of 0%, 5%, 10%, 15%, or 20% *w/w* trehalose solution. The nanoparticle suspensions were lyophilized, redispersibility was evaluated, and particle size of the resuspended nanoparticles was measured.

### 3.7. Lyophilization

Progesterone-loaded stearic acid SLNs suspended in 20% trehalose solution were placed into glass jars at a depth of approximately 1 cm, tightly capped with a metal screw cap and placed in the freezer overnight. The lyophilizer (HarvestRight 4-shelf bench top freeze dryer, HarvestRight, North Salt Lake, UT, USA) was then prechilled to a −25 °C shelf temperature (−50 °C condenser temperature) and the jars were opened and loaded. The automated drying cycle included 6 h at a shelf temperature of −28 °C and a vacuum of less than 100 microns, followed by 42 h at a shelf temperature of −14 °C again at a vacuum of less than 100 microns.

### 3.8. Determination of Entrapment Efficiency and Drug Loading

The drug loading and entrapment efficiency was determined by quantifying the amount of progesterone in the freeze-dried SLN formulation using high performance liquid chromatography (HPLC). The following formulae were used to calculated drug loading and entrapment efficiency (Equations (1) and (2)).

Drug loading = (total entrapped drug/total nanoparticle weight) × 100(1)

Entrapment efficiency = ((amount of total drug − free drug)/amount of total drug) × 100(2)

### 3.9. HPLC Analysis and Sample Preparation

#### 3.9.1. HPLC Conditions

Quantification of progesterone was performed using the method of Pareira [18] with modification using a reversed-phase HPLC system with a PDA detector (Waters 2695 and 996, Waters Assoc, Milford Mass, MA, USA), with a C-18 column (30 cm × 4.6 mm, 5 µm, Zorbax RX-C18, Agilent, Santa Clara, CA, USA). The mobile phase was 80:20 methanol/water at a flow rate of 1.0 mL/min. Detection was performed at 240 nm (the approximate lambda max of progesterone). Retention time for progesterone was approximately 6.8 min (k’ = 2.4).

Standard solutions were prepared by dissolving progesterone in mobile phase and sonicating for 15 min. Calibration standards were prepared by diluting standard solutions with mobile phase. Calibration curves were prepared ranging from 3 to 200 µg/mL. Daily standards were run to confirm system suitability.

#### 3.9.2. Sample Preparation

Liquids: Raw SA-P SLNs and centrifuged supernatant were prepared by dissolving 0.5 mL suspended solids in 2 mL THF, sonicating for 15 min. This resulted in a particle-free solution and was injected directly into the HPLC.

Solids: Then, 20–30 mg solid pellet (without trehalose) was dissolved with 2 mL of THF and sonicated for 15 min, resulting in a clear solution. This was injected into the HPLC. Solid freeze-dried SLNs (with trehalose) were prepared by dispersing 20–30 mg of solid in 2 mL of THF and sonicating for 15 min. After sonication, there were still visible suspended particles in the mixture, presumably trehalose. Eight mL of mobile phase (80:20 methanol/water) was added to the sample and sonicated for another 15 min, resulting in a clear solution. This sample was injected into the HPLC.

### 3.10. Differential Scanning Calorimetry

Differential scanning calorimetry (DSC) was performed on solid samples of progesterone, stearic acid, trehalose, and progesterone-loaded stearic acid SLNs (Diamond DSC, Perkin Elmer, Waltham, MA, USA). Data were collected and analyzed via PYRIS software (version 13, Perkin Elmer, Waltham, MA, USA). Approximately 5 mg of solid sample was placed in an aluminum sample pan and crimped closed for analysis. Samples were held isothermally at 40 °C and then heated to at least 200 °C at a 20 °C/min scanning rate.

### 3.11. Polarized Light Microscopy

Samples were visualized using AmScope polarized light microscope at either 100× or 500× magnification, and images were captured using an 18-megapixel digital camera via AmScope 3.7 digital imaging software (AmScope, Irvine, CA, USA).

### 3.12. Short Term Stability of Progesterone-Loaded SLNs Formulated with 20% Trehalose

A short-term (4-week) evaluation of the stability of progesterone–stearic acid SLNs after they were lyophilized in a 20% trehalose solution was conducted at two conditions: 25 °C/ambient (50%) relative humidity and 40 °C/25% relative humidity. SLN–trehalose SLN samples were lyophilized in glass jars, and once removed from the lyophilizer all samples (except time-zero) were immediately closed with a screw-top cap and placed in stability chambers. When samples were to be analyzed they were allowed to come to room temperature, opened, and sampled for assay and the remaining sample was reconstituted with the amount of water (mL/g dried solid) that it had lost during lyophilization. This reconstituted mixture was evaluated for dispersibility.

## 4. Results and Discussion

### 4.1. Progesterone Solubility in SA

Molten stearic acid at 80 °C is colorless and completely transparent, allowing for direct observation of the solubilization of progesterone. Progesterone was added to 28% *w/w*, and after addition to 30% *w/w* of progesterone, the mixture remained opaque, indicating a saturated solution of the active in stearic acid had been achieved.

### 4.2. Selection of Surfactants and Concentration

The lecithin/sodium taurocholate system was chosen as it had the smallest particle size and greatest zeta potential (Table 1). Zeta potential was being investigated for stability purposes. A zeta potential greater than –30 mV or +30 mV suggests good stability [19] and in general, a large zeta potential is desired to avoid agglomeration [20]. The lecithin/sodium taurocholate system was the only surfactant system with a zeta potential greater than –30 mV so it was expected to be the most resistant to agglomeration over time. Two percent surfactant concentration was selected based on the most consistent mean particle size between measurements (Table 2).

### 4.3. Ultracentrifugation Optimization

**Figure 1:**
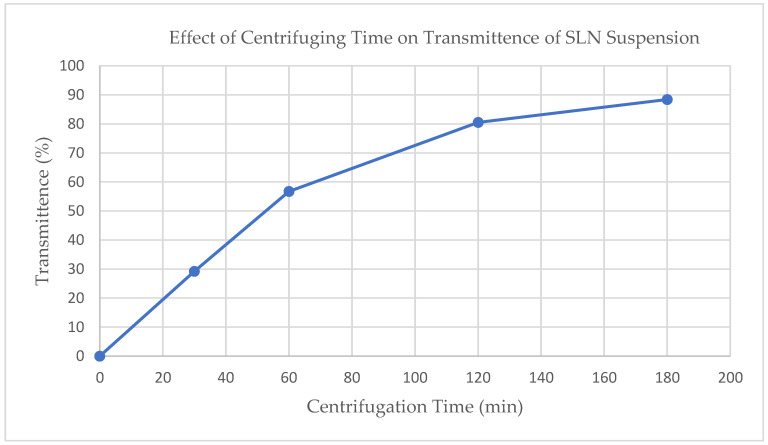
Effect of ultracentrifugation time on SLN suspension on light scattering at 850 nm.

The extent of ultracentrifugation at 15,000 RPM (RCF = 40,000) was monitored and controlled using visible light transmittance at 850 nm (see Figure 1). Based on these results, an ultracentrifugation time of 120 min was chosen balancing process time with extent of separation.

### 4.4. Determination of Entrapment Efficiency and Drug Loading

Drug loading and entrapment efficiency were calculated according to Equations (1) and (2) based on analytical measurements of progesterone content in dried lyophilized cake and ultracentrifuged supernatant. Drug loading was measured to be 4.11% (SD = 0.388%, *N* = 3), while entrapment efficiency was determined to be 96.1% (sd = 3.35%, *N* = 3), both after adjusting for a 6% loss in water due to evaporation in the emulsion process. Progesterone recovered from the initial ultracentrifugation step was approximately 3.4%, while that recovered from the wash step was only found to be 1.3%, suggesting the wash step may not be required for future preparations using this process. Such a high entrapment efficiency and low aqueous supernatant recovery may be anticipated based on the comparison of the aqueous solubility of progesterone with its solubility in molten stearic acid.

### 4.5. Differential Scanning Calorimetry

**Figure 2:**
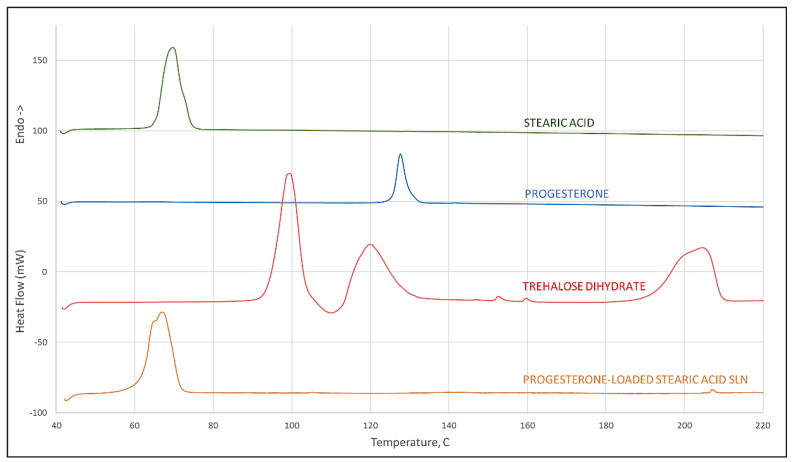
DSC scan of stearic acid, progesterone, trehalose dihydrate (peak at 93 °C corresponds to dehydration of the dihydrate crystal, peak at 115 °C to the melt of the α-anhydrate, and 190 °C to melting of the β-anhydrate crystal), and progesterone-loaded stearic acid SLNs.

Results of differential scanning calorimetry evaluations are given in Figure 2. Scans of stearic acid, progesterone, and trehalose dihydrate are given individually showing melting endotherms of 65 °C and 126 °C, respectively, for stearic acid and progesterone, and a treholose dihydrate dehydration endotherm at 93 °C signifying its transition to trehalose α-anhydrate. From its thermogram it can be observed that trehalose has an endothermic peak at approximately 115 °C which corresponds to the melt of the α-anhydrate, and another at 190 °C corresponding to the melt of the β-anhydrate. These observations for trehalose are all in agreement with the recent literature [21]. A scan of progesterone-loaded stearic acid SLNs shows a shift to a slightly lower temperature of the stearic acid melt with a shoulder that is not evident in the stearic acid melt and that shows no evidence of a progesterone melt. This provides strong evidence of interaction between progesterone and stearic acid [22].

### 4.6. Polarized Light Microscopy

**Figure 3:**
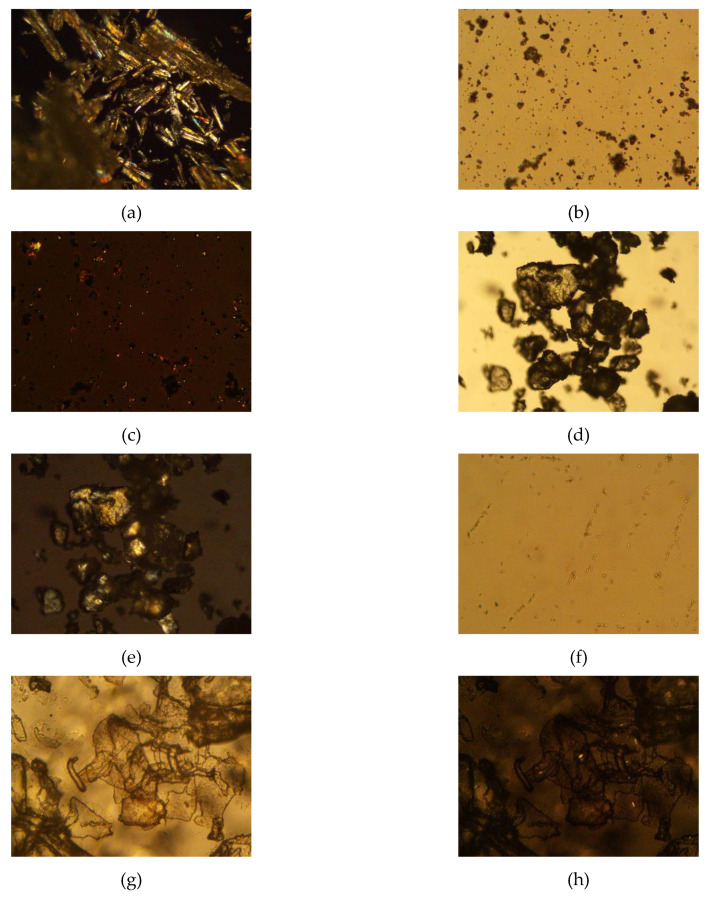
Photomicrographs of raw materials and processed samples; (**a**) stearic acid raw material, 100x, polarized light (crossed polars); (**b**) progesterone raw material, 500×, non-polarized light (open polars) and (**c**) progesterone raw material, 500×, polarized light (crossed polars); (**d**) trehalose didydrate raw material, 100×, non-polarized light (open polars) and (**e**) trehalose didydrate raw material, 100×, polarized light (crossed polars); (**f**) progesterone-loaded stearic acid SLNs, 500×, polarized light (crossed polars); (**g**) progesterone-loaded stearic acid SLNs lyophilized in 5% trehalose solution, 500×, non-polarized light (open polars) and (**h**) progesterone-loaded stearic acid SLNs lyophilized in 5% trehalose solution, 500×, polarized light (crossed polars).

**Figure 4:**
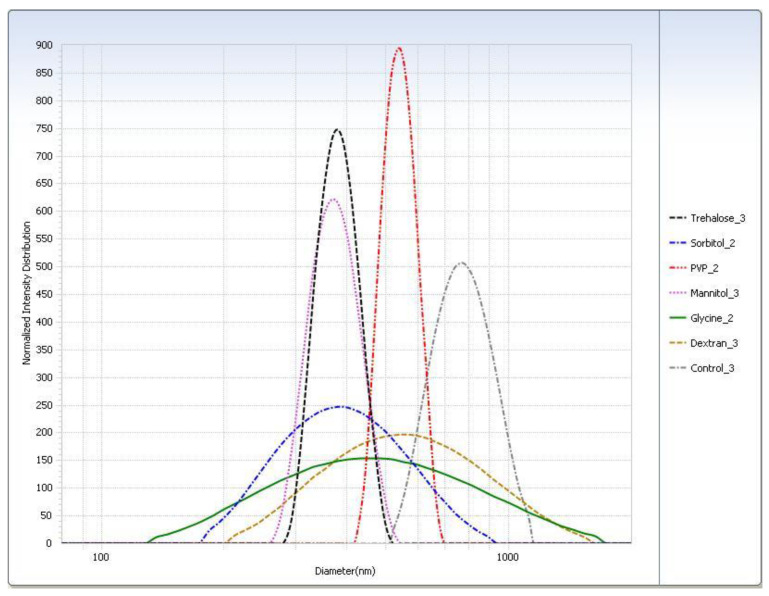
Intensity distribution of cryoprotected SA-P SLNs after freeze–thaw study at the −20 °C (slow freeze) condition.

Stearic acid, progesterone, trehalose, and progesterone-loaded stearic acid SLNs dried with and without trehalose were observed using polarized light microscopy (Figure 3). Stearic acid shows a strong birefringence, while progesterone and trehalose are somewhat less birefringent. Progesterone-loaded stearic acid particles are shown in Figure 4. Particles were visible down to approximately 1 µm in size. Based on particle size analysis, it is fully anticipated that smaller SLNs are present in this sample as well. Visible particles appear to be birefringent similar to stearic acid, suggesting that these and smaller particles are in fact predominantly stearic acid. Based on our calculated entrapment efficiency, progesterone is entrapped in these particles as well. When SLNs were mixed with trehalose and lyophilized, the resulting particles show a particularly non-birefringent character, in contrast to trehalose raw material, supporting the conclusion that progesterone-loaded stearic acid SLNs are encased in trehalose glass indicating successful lyophilization [23].

### 4.7. Effect of Lyophilization on Progesterone-Loaded SLNs

#### 4.7.1. Selection of Cryoprotectant

For the freeze–thaw study (Table 3), in general the rapid freeze condition yielded smaller particle sizes than the slow freeze condition. The PI was smaller for almost all cryoprotectants in the rapid freeze condition. The particle sizes measured for the trehalose, sorbitol, and mannitol suspensions were similar. Glycine displayed the largest difference in particle size of SLNs after thawing between the two freezing conditions. Particle size distributions are shown graphically in Figure 4.

**Figure 5:**
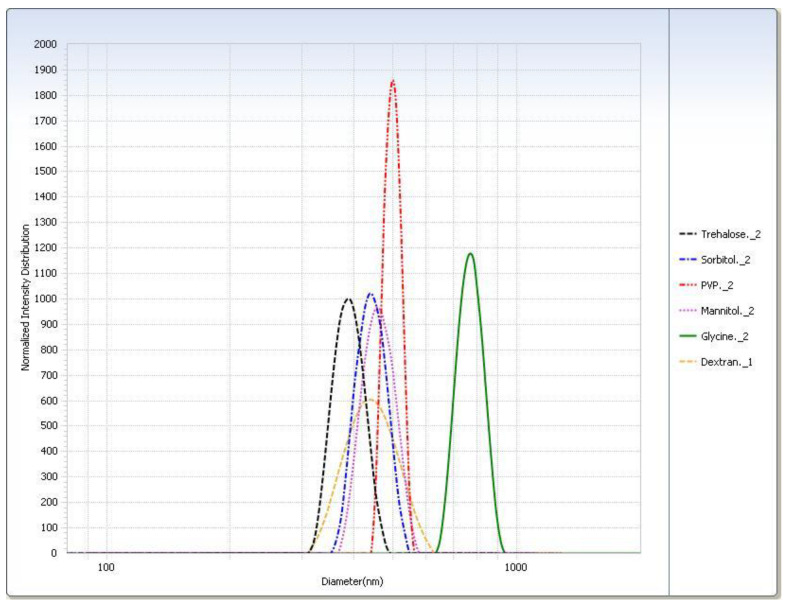
Intensity distribution of cryoprotected SA-P SLNs after lyophilization at the −20 °C (slow freeze) condition.

After lyophilization, the control sample was poorly redispersed regardless of freezing condition so much so that the particle size could not be measured (Table 4). With the exception of the slow freeze PVP, all lyophilized cryoprotectant samples were readily redispersed. Trehalose had the smallest particle size after redispersion (Table 5). All samples had shown considerable particle growth, and as with the freeze–thaw study, glycine displayed the largest difference between freezing conditions. Trehalose was selected as the cryoprotectant as it had the smallest particle size (Figure 5) and was easily redispersed (Table 6). Results from the −20 °C (slow freeze) condition were of particular interest because this condition is expected to result in larger ice crystals than faster freezing conditions [24] with the larger ice crystals resulting in higher solute and suspension concentrations in the unfrozen phase in which trehalose and SLNs reside [12]. It is felt that successful results at a slow freezing condition are indicative of a more robust cryoprotection.

#### 4.7.2. Optimization of Cryoprotectant Concentration

Consistent with previous results, the control sample could not be redispersed or analyzed for particle size (Table 7). All concentrations of trehalose were easily redispersed. The 20% trehalose sample demonstrated the smallest particle size and PI (Table 8). Based on this outcome, 20% trehalose was chosen as the optimal cryoprotectant concentration.

#### 4.7.3. Mechanisms of Trehalose Cryoprotection

Trehalose functions as a cryoprotectant during freezing above Tg’ (the glass transition temperature of the maximally freeze-concentrated solute), where the trehalose–water solution exists as a viscous dispersion, and below Tg’ during primary drying, where the trehalose–water solution exists as a glassy matrix. As trehalose solution solidifies during the freezing process, ice crystals form in equilibrium with freeze-concentrated trehalose–water solution, in which colloidal SA-P SLNs are suspended. As temperature continues to fall, approaching the Tg’ of trehalose–water (−29 °C) [25], the concentration of trehalose reaches approximately 80% [25], creating a high viscosity suspension medium for the SLNs. This high viscosity reduces the interactions between nanoparticles and thereby reduces the likelihood of agglomeration [17]. Furthermore, the volume in which SLNs exist in suspension at Tg’ is proportional to the initial preparation concentration of trehalose, suggesting a higher concentration of cryoprotectant will ensure lower interaction of SLNs during the freeze-concentration process [17]. Once the temperature of the ice/freeze-concentrated trehalose–water system is taken below the Tg’, a glassy matrix is formed which immobilizes and protects the nanoparticles from further interaction [12,26].

### 4.8. Short Term Stability of Progesterone-Loaded SLNs Formulated with 20% Trehalose

Table 9 shows the result of a short-term evaluation of the stability of progesterone–stearic acid SLNs lyophilized with 20% trehalose. Results show that there is some additional agglomeration of SLNs on stability at both 25 °C/50% RH and 40 °C/25% RH based on respective increases in both particle size and PI measurement. This may be due to the presence of moisture at both stability conditions and the deliberate packaging in moisture-permeable containers. Storage at 40 °C showed a drop in content of progesterone at 1, 2, and 4 weeks, indicating a possible chemical degradation at the higher storage temperature in addition to likely conversion of trehalose anhydrate to dihydrate. These results would suggest storage at refrigerated conditions with protection from moisture would be appropriate. Because it was anticipated that the glass jar and closure used to package the lyophilized cake were not moisture-tight, lower humidity conditions than the regulatory authority-standard 25 °C/60% RH and 40 °C/75% RH were employed.

## 5. Conclusions

A formulation of SA-P SLNs was optimized with respect to the type and concentration of the surfactant and cryoprotectant. Progesterone solubility in molten stearic acid (80 °C) was found to be 28% *w/w*, far exceeding the formulation level of 9.1% *w/w* (progesterone in stearic acid). Ultracentrifugation of the SLNs at a fixed rotor speed (15,000 rpm) was investigated and a preferred time of 120 min was selected. This investigation has reported the effect of surfactant type and concentration on the particle size, size distribution, and zeta potential and the best surfactant found in the group studied was lecithin/sodium taurocholate at 2% *w/w*. Lyophilization of redispersed SA-P SLNs was shown to cause significant agglomeration. Lyophilization studies showed a trehalose level to provide the best inhibition of particle size growth. The findings of this study confirm the need for use of a cryoprotectant in the lyophilization of SA-P SLNs, the selection of an appropriate cryoprotectant, and the selection of an optimized cryoprotectant concentration. 

## Figures and Tables

**Table 1:** Particle size and zeta potential of surfactant systems; data presented as mean (standard deviation), *N* = 3.

Surfactant (0.5%)	Particle Size Mean (SD) (nm)	PI Mean (SD)	Zeta Potential Mean (SD) (mV)
Sodium taurocholate (0.25%) and Lecithin (0.25%)	315.2 (5.534)	0.001937 (0.000259)	−43.26 (1.44)
Poloxamer 188	319.6 (6.951)	0.005854 (0.004919)	−10.18 (3.67)
Kolliphor RH 40	374.0 (8.693)	0.02158 (0.01198)	−17.76 (5.58)
Tween 80	346.4 (3.988)	0.08224 (0.04563)	1.24 (1.29)

**Table 2:** Effect of surfactant concentration on particle size and zeta potential; data presented as mean (standard deviation), *N* = 3.

Surfactant Concentration (%)	Particle Size Mean (SD) (nm)	PI Mean (SD)	Zeta Potential (mV) Mean (SD)
0.5	315.2 (5.534)	0.001937 (0.000259)	−43.26 (1.44)
1	397.6 (10.70)	0.027603 (0021363)	−32.68 (2.16)
2	385.7 (0.07506)	0.002161 (0.00000567)	−45.52 (1.22)

**Table 3:** Freeze–thaw study: Effect of cryoprotectant on the particle size and distribution of SLNs immediately after thawing; data presented as mean (standard deviation), *N* = 3.

Sample (−20 °C/Slow Freeze)	Particle Size Mean (SD) (nm)	PI Mean (SD)	Sample (−70 °C /Rapid Freeze)	Particle Size Mean (SD) (nm)	PI Mean (SD)
Control	726.5 (106.5)	0.023 (0.0050)	Control	596.5 (28.60)	0.00287 (0.000415)
Dextran	514.9 (8.822)	0.103 (0.0787)	Dextran	487.6 (25.75)	0.00460 (0.00201)
Glycine	431.7 (5.493)	0.217 (0.157)	Glycine	355.2 (14.25)	0.0102 (0.00674)
Mannitol	379.1 (6.213)	0.014 (0.00388)	Mannitol	381.1 (13.71)	0.00924 (0.00361)
PVP	528.6 (45.92)	0.0012 (0.00792)	PVP	513.7 (38.48)	0.00757 (0.00467)
Sorbitol	374.6 (14.70)	0.102 (0.0833)	Sorbitol	360.4 (19.07)	0.0119 (0.00737)
Trehalose	374.1 (8.100)	0.0265 (0.0193)	Trehalose	358.8 (3.821)	0.0652 (0.0618)

**Table 4:** Freeze–thaw study: Effect of cryoprotectant on the redispersibility of SLNs on reconstitution after lyophilization. Redispersion qualitative scale: 1—fully redispersed within 30 s of vortexing; 2—a few remaining undispersed visible agglomerates still present after 30 s of vortexing; 3—very little or no redispersion of solid after 30 s of vortexing.

Sample	Redispersion Quality
Control 20	3
Dextran 20	1
Glycine 20	1
Mannitol 20	1
PVP 20	2
Sorbitol 20	1
Trehalose 20	1
Control 70	3
Dextran 70	1
Glycine 70	1
Mannitol 70	1
PVP 70	1
Sorbitol 70	1
Trehalose 70	1

**Table 5:** Freeze–thaw study: Effect of cryoprotectant on the particle size and distribution of SLNs on reconstitution after lyophilization (*N* = 3).

Sample (−20 °C /Slow Freeze)	Particle Size Mean (SD) (nm)	PI Mean (SD)	Sample (−70 °C /Rapid Freeze)	Particle Size Mean (SD) (nm)	PI Mean (SD)
Control *	-	-	Control *	-	-
Dextran	466.2 (14.84)	0.044 (0.033)	Dextran	445.8 (19.08)	0.0026 (0.00067)
Glycine	930.6 (102.6)	0.0062 (0.0044)	Glycine	1292.2	0.0062 (0.000089)
Mannitol	523.2 (34.79)	0.0077 (0.00049)	Mannitol	705.9	0.0027 (0.00043)
PVP	537.8 (37.35)	0.0026 (0.00061)	PVP	488.0	0.0026 (0.00050)
Sorbitol	448.2 (13.04)	0.00037 (0.00026)	Sorbitol	420.5	0.0044 (0.0011)
Trehalose	374.0 (8.100)	0.0063 (00036)	Trehalose	397.2	0.0035 (0.0022)

* sample could not be redispersed for particle size analysis.

**Table 6:** Freeze–thaw study: Effect of cryoprotectant on redispersibility of SLNs after lyophilization. Redispersion qualitative scale: 1—fully redispersed within 30 s of vortexing; 2—a few remaining undispersed visible agglomerates still present after 30 s of vortexing; 3—very little or no redispersion of solid after 30 s of vortexing.

Sample	Redispersion Quality
Control 20	3
Dextran 20	1
Glycine 20	1
Mannitol 20	1
PVP 20	2
Sorbitol 20	1
Trehalose 20	1
Control 70	3
Dextran 70	1
Glycine 70	1
Mannitol 70	1
PVP 70	1
Sorbitol 70	1
Trehalose 70	1

**Table 7:** Effect of trehalose concentration of the redispersibility of SLNs after lyophilization. Redispersion qualitative scale: 1—fully redispersed within 30 s of vortexing; 2—a few remaining undispersed visible agglomerates still present after 30 s of vortexing; 3—very little or no redispersion of solid after 30 s of vortexing.

Percent Trehalose	Redispersion Quality
0%	3
5%	1
10%	1
15%	1
20%	1

**Table 8:** Effect of trehalose concentration on particle size and distribution of SLNs after lyophilization.

Percent Trehalose	Particle Size Mean (SD) (nm)	PI Mean (SD)
0%	Unable to redisperse	Unable to redisperse
5%	376.7 (4.371)	0.044 (0.022)
10%	361.6 (5.412)	0.027 (0.0072)
15%	374.1 (14.76)	0.11 (0.066)
20%	332.5 (10.65)	0.0061 (0.0021)

**Table 9:** Summary of short-term stability study of progesterone-loaded stearic acid SLNs. Redispersion qualitative scale: 1− fully redispersed within 30 s of vortexing; 2—a few remaining undispersed visible agglomerates still present after 30 s of vortexing; 3− very little or no redispersion of solid after 30 s of vortexing.

Storage Time	Mean Assay, % Progesterone *w/w* (*N* = 2)	Particle Size Mean (SD) (nm)	PI Mean (SD)	Redispersibility
Initial	1.97	319.8 (1.762)	0.0044 (0.0035)	1
25 °C/50% RH				
2 weeks	1.94	667.7 (65.47)	0.0059 (0.0059)	1
4 weeks	1.85	531.0 (19.92)	0.15 (0.12)	1
40 °C/25% RH				
1 week	1.42	425.6 (13.42)	0.0081 (0.0046)	1
2 weeks	1.44	663.9 (21.80)	0.010 (0.0076)	1
4 weeks	1.45	695.3 (32.66)	0.57 (0.27)	1
